# Clinical characteristics and biopsy accuracy in suspected cases of Sjögren’s syndrome referred to labial salivary gland biopsy

**DOI:** 10.1186/s12891-015-0482-9

**Published:** 2015-02-15

**Authors:** Raquel A Giovelli, Maria CS Santos, Érica V Serrano, Valéria Valim

**Affiliations:** Medical Clinic Department, Center of Health Science, Federal University of Espírito Santo, Vitória, Brazil; Pathology Department, Center of Health Science, Federal University of Espírito Santo, Vitória, Brazil; Universitary Hospital Cassiano Antônio de Moraes, Av. Marechal Campos, 1460, CEP 29040-090 Vitória, Brazil

**Keywords:** Biopsy, Labial salivary gland, Primary Sjögren’s syndrome, Sensibility, Specificity

## Abstract

**Background:**

Labial salivary gland biopsy (LSGB) is the most important diagnostic tool for the diagnosis of Sjögren’s syndrome (SS), but its diagnostic value is rarely studied. This study assessed the sensibility and specificity of LSGB, and the clinical profiles of patients who were referred for biopsy.

**Methods:**

Retrospective analysis of the histopathological reports from LSGB and medical report data from patients who underwent LSGB between 2008 and 2011 was conducted.

**Results:**

About 290 biopsies were performed and 74 were excluded due to insufficient clinical data. Of the 216 patients, 0.46% was carrier of hepatitis C virus, 30.1% had primary SS (pSS), and 8.8% had secondary SS (sSS). Of the samples, 94.3% presented dryness symptoms, 51.6% experienced dryness only, 42.7% had systemic manifestations, and 66.9% presented low unstimulated salivary flow and/or Schirmer’s test. LSGB was necessary in 67.6% to confirm the presence of SS based on the American-European Consensus Group 2002 criteria (AECG). Based on specialist’s opinion, sensibility level was 86.57%, and specificity was 97.43%. Positive predictive value (PPV) was 95%, and negative predictive value (NPV) was 92.6%. Determined accuracy was 93.3%. Concordance (kappa coefficient) of LSGB and specialist’s opinion was 0.851, and LSGB with AECG criteria was 0.806. Of the 98 patients referred with fibromyalgia and dryness, 36.7% had SS and LSBG focus score of ≥ 1. Patients with SS were older, and showed more severe lachrymal and salivary dysfunctions, greater frequency of fibromyalgia, anti-nuclear antibodies (ANA), anti-SSA-Ro, and anti-SSB-La.

**Conclusions:**

Labial salivary gland biopsy has high sensibility, specificity, positive and negative predictive values for diagnosis of pSS. In the clinical practice, it is useful, especially for those patients with glandular dysfunctions and negative antibodies.

## Background

Sjögren’s syndrome (SS) is considered the second most common rheumatic autoimmune disease affecting between 0.05% and 0.4% of the world population [1-3]. In spite of being relatively common, it is still rarely diagnosed because of a pleomorphic presentation varying from mild cases of dryness, fatigue, and pain confounding with fibromyalgia (FM) to severe systemic cases similar to rheumatoid arthritis (RA) and systemic lupus erythematosus (SLE). It also has a wide differential diagnosis including infection by hepatitis C and HIV, hyper IgG4 syndrome, sarcoidosis, and lymphoma [4].

The difficulty in diagnosis is reflected on continuous review attempts of the 7 classification criteria that have been created in the past 25 years [5]. However, histological analysis of labial salivary gland biopsy (LSGB) is mostly a method of great importance according to the American-European Group Consensus (AEGC) criteria [6] and the criteria proposed by the American College Rheumatology in 2012 (ACR 2012) [7]. The indication for the LSGB performance has not been well established yet in clinical practice, and few published studies have evaluated the sensibility and specificity of LSGB in primary SS (pSS) [8]. Only two studies identified patients with and without pSS not using LSGB findings and clinical re-evaluation (specialist’s opinion) [9,10]. Other studies have used AECG, but it presents bias because LSGB is part of the AECG criteria [11-17]. A recent systematic review indicated a lack of information about the diagnostic value of MSGB [8].

The main objective of this study was to evaluate the biopsy accuracy based on suspected cases of SS referred to LSGB. Also, we described the clinical characteristics and glandular dysfunctions of patients referred to biopsy, comparing patients with pSS and nonspecific dryness syndrome.

## Methods

This was a retrospective study including all patients from the Rheumatology Unit of the University Hospital of the Federal University of Espírito Santo (HUCAM/UFES/EBSERH), above 18 years old, and referred for LSGB to investigate SS between March 2008 and March 2011. All recorded histological reports of LSGB in that period were assessed.

### Labial salivary gland biopsy technique and histological parameters

LSGBs were performed by 2 experienced rheumatologists, using linear incision [18] described as follows. Lidocaíne 2% with 1 ml of epinephrine was injected in the mentonian foramen to block nerve. The lower lip was everted to find a normal and healthy area, usually the left side when possible. The best site was chosen by palpation to find glands. A horizontal incision as minimal as possible, usually less than 1 cm, was made over the gland by scalpel (blade 3). Simultaneously, the outside lip was compressed to improve hemostasia. Glands usually bulged from the wound. The wounds were moved and rolled to expose the glands better. Glands (4–8) were collected carefully to avoid harming the vessels and nerves. One to three surgical stitches using silk or resorbable suture were necessary.

Histopathological analysis was performed by 2 experienced pathologists. They scored the focus numbers and have considered compatibility with SS if the focus score is ≥ 1 (“positive biopsy”), based on the classification described previously [18-20].

### Dryness symptoms and glandular dysfunction

Concurrently with biopsy, patients performed Schirmer’s test I (without anesthesia) (ST) and unstimulated salivary flow (USF). Glandular dysfunction was defined if ST was < 5 mm in 5 minutes using standardized sterilized test strips (Ophtalmos, São Paulo, Brazil) and/or USF was ≤ 1.5 ml/min measured in 15 minutes. Patients were recommended to have breakfast without coffee or chocolate. All measures were done between 8 a.m. and 9 a.m.

Just before glandular function evaluation, patients answered some questions to identify dryness symptoms [6]:

1. Have you had daily, persistent, troublesome dry eyes for more than 3 months?
2. Do you have a recurrent sensation of sand or gravel in the eyes?
3. Do you use tear substitutes more than 3 times a day?
4. Have you had a daily feeling of dry mouth for more than 3 months?
5. Have you had recurrently or persistently swollen salivary glands as an adult?
6. Do you frequently drink liquids to aid in swallowing dry food?

### Clinical parameters

Demographic, autoantibodies, clinical manifestations, comorbidities, and reasons for being referred for LSGB were obtained through medical reports. The autoantibodies evaluated were antinuclear antibody (ANA) using indirect immunofluorescence, rheumatoid factor (RF) measured by turbidimetry, and anti-SSA-Ro and anti-SSB-La using hemagglutination.

Patients were classified as having SS according to the AECG criteria and specialist’s opinion. The specialist’s opinion was maiden considering the diagnosis described in the medical report and re-evaluation by 2 specialists.

### Statistical analysis and ethical aspects

Patients with insufficient data for SS diagnosis or withdrawers were not included in the analysis.

Demographics, reasons for biopsy indication, clinical manifestation, and glandular dysfunction of the referred patients for LSGB were described.

Sensibility, specificity, positive predictive value (PPV), and negative predictive value (NPV) of LSGB were calculated with the specialist’s opinion serving as the gold-standard.

Patients were also classified according the AECG criteria [6], and comparisons between patients with primary SS and nonspecific dryness syndrome were evaluated in terms of demographics, comorbidities, glandular dysfunction, and presence of auto-antibodies.

Concordance levels between LSGB and specialist’s opinion, and LSGB and AECG were calculated by the Kappa coefficient.

The collected data were analyzed and processed using the IBM SPSS Statistical Package for Social Sciences version 19 (IBM, Armonk, New York, USA). Mann–Whitney and Z (Chi-square) tests were used for the comparison between patients with and without SS. It was considered significant when the p value is < 0.05.

This Project was approved by the Ethics Committee in Research of the Health Science Center from the Federal University of Espírito Santo in October 26, 2011 (protocol number 241/11). Written informed consent for participation in the study were not required by Ethics Committee because it was a retrospective study, and all procedures were routine for SS diagnosis. We have obtained a written consent by Hospital director to access all registered information in the hospital system.

## Results

Two-hundred-ninety (290) individuals underwent LSGB during the period to investigate SS. Seventy-four patients (74) were excluded due to lack of clinical information. Demographic and clinical characteristics of the 216 included individuals are detailed in Table 1. All biopsies had sufficient salivary gland samples (≥4 mm^2^), and only 7 cases < of 8 mm^2^. The average was 12.52 ± 5.30 mm^2^.

**Table 1:** **Demographic and clinical characteristics of the suspected cases referred for labial salivary gland biopsy**

**Parameters**	**N = 216**
Age (years)	47.7 ± 12.5
Gender (Women)	188 (87%)
Dryness symptoms (only)	111 (51.4%)
Dryness and systemic symptoms	92 (42.6%)
Systemic symptoms (only)	12 (5.55%)
Virus C Hepatitis	1 (0.45%)
Unstimulated salivary flow (<0.1 ml/min)	119 (55.09%)
Schirmer test I (≤5 mm)	103 (47.68%)
Anti-SSA and/or anti-SSB	36 (16.66%)
ANA	100 (46.29%)
Rheumatoid Factor (latex)	40 (18.51%)
Focal Sialoadenitis (focus score ≥ 1)	79 (36.57%)

ANA = antinuclear antibodies.

Dryness was the reason to investigate SS in 94.3% of the samples. About 51.6% had dryness only, and 42.7% had associated extra-glandular manifestations. Only 5.5% of the patients were referred because of extra-glandular manifestation without dryness, with 2 having polyneuropathy, 1 had positive ANA and polyarthralgia, 3 had positive ANA and polyarthritis, 1 had central nervous system vasculitis, 1 had recurrent parotiditis, 1 had rheumatic fever with mitral regurgitation, 1 had scleritis, 1 had pancytopenia, 1 had polyarthritis that was defined as rheumatoid arthritis afterwards. Of the patients referred for biopsy, 66.9% (N = 144) showed ST of < 5 mm and/or USF of ≤ 1.5 ml/min.

To analyze biopsy accuracy for primary SS diagnosis, we included 183 patients. Nineteen (19) with secondary SS and 13 without the specialist’s opinion in the medical report were excluded from a total of 216. Biopsy was positive (≥1 focus score) in 61 patients (58 having SS according to specialist’s opinion, and 3 not having SS). Biopsy was negative (<1 focus score) in 122 (9 having SS according to specialists opinion and 113 not having SS). Sensibility level was 86.57%, and specificity was 97.43%. The positive predictive value (PPV) was 95%, and the negative predictive value (NPV) was 92.6%. Determined accuracy level was 93.3%.

**Figure 1:**
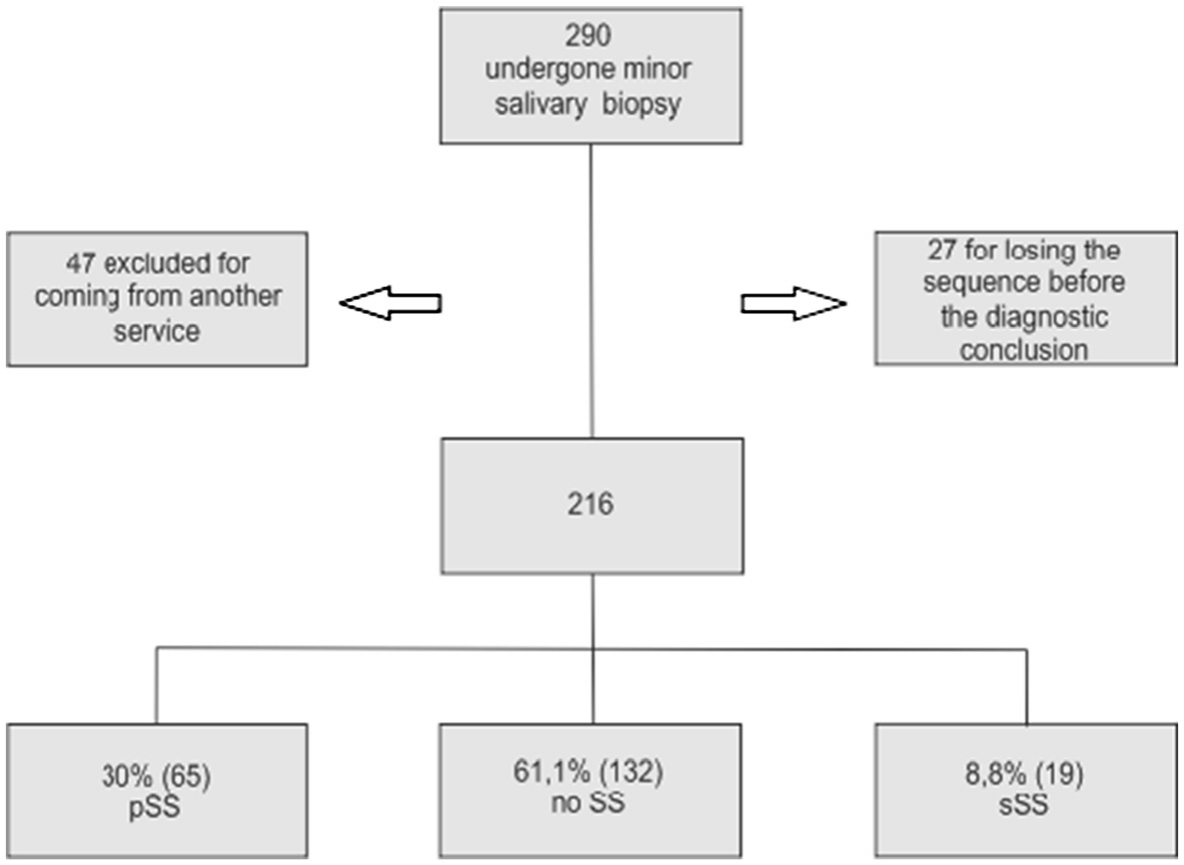
**Flow chart study.** pSS: primary Sjögren’s syndrome according to AECG, sSS: secondary Sjögren’s syndrome according to AECG, 8 SLE: Systemic Lupus Erythematosus, 6 RA: Rheumatoid Arthritis, 3 Overlap (SSc: Systemic Sclerosis, APS: Antiphospholid Syndrome, SS: Sjögren’s syndrome).

Patients were also classified according to the AECG (Figure 1). Concordance of LSGB was high and significant (p < 0.05) with both, but higher with specialist’s opinion (kappa = 0.851) than AECG criteria (kappa = 0.806).

**Figure 2:**
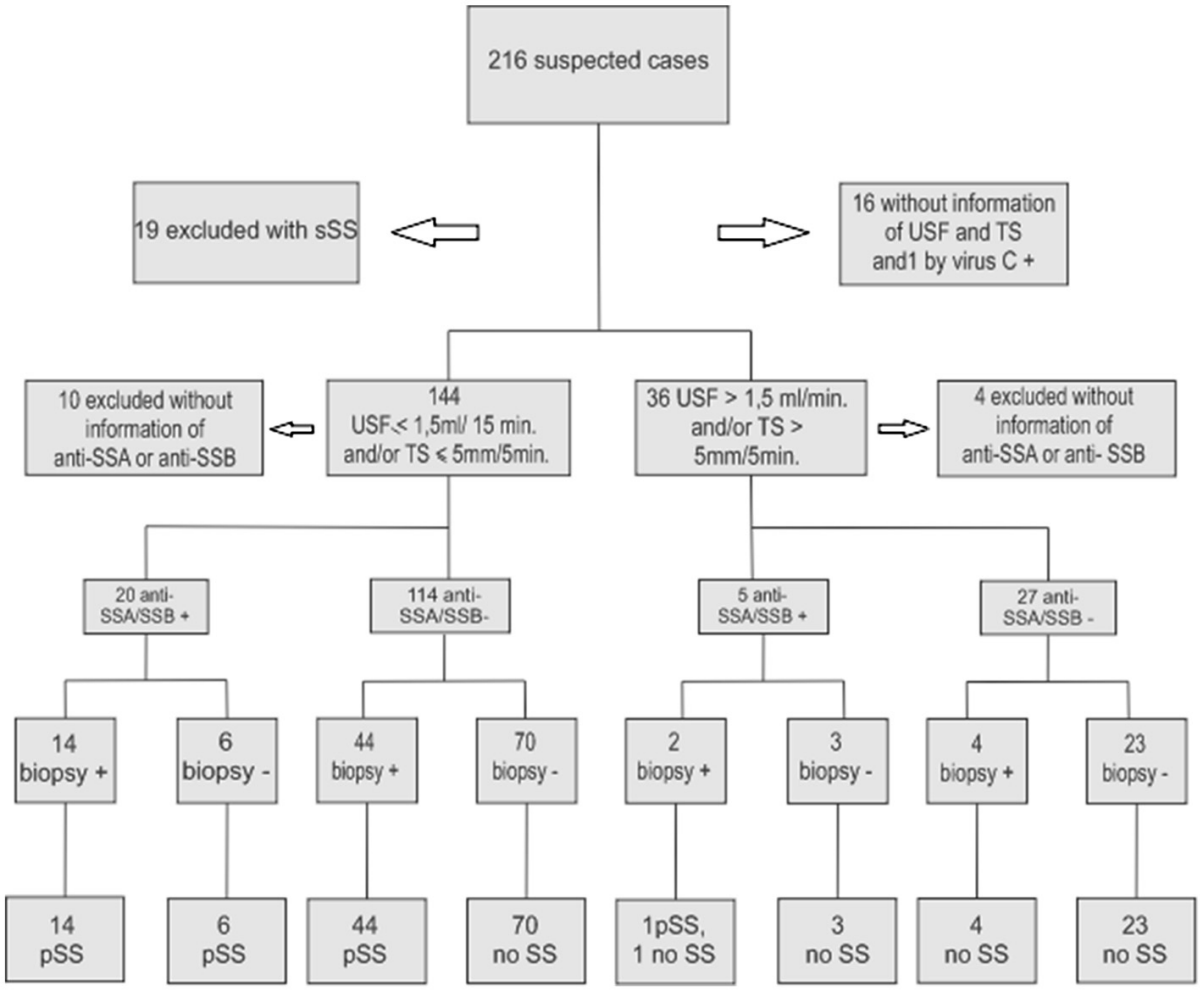
**Diagnostic flow of suspected patients of Sjögren’s syndrome.** USF: unstimulated salivary flow; ST: Schirmer test; anti-SSA/SSB +: anti-SSA and/or anti-SSB positive; anti-SSA/SSB -: anti-SSA and/or anti-SSB. negative; Biopsy +: score focus ≥ 1; Biopsy -: focus score < 1; no SS: no Sjögren’s. syndrome; pSS: primary Sjögren’s syndrome according to the American European. Consensus Group 2002.

LSGB was necessary in 67.6% (n = 44) to fulfill the AECG criteria for SS. The results of the investigation flow for the diagnosis of SS is shown in Figure 2. The combination of glandular dysfunction and positive serology (anti-SSA-Ro and/or anti-SSB-La) seemed to be useful in identifying positive biopsy and SS diagnosis. Most patients (N = 14, 70%) with ST of <5 mm/min and/or USF of ≤1.5 ml/min had LSGB compatible with SS and met the AECG criteria for pSS. On the other hand, great majority (N = 23, 85.1%) of patients with no lachrymal and salivary dysfunction, besides negative antibody, had LSGB focus score of < 1, and no one fulfilled the AECG criteria for SS.

In spite of being common autoantibodies in SS, the presence of ANA ≥ 1/320 and positive RF concomitantly with negative anti-SSA-Ro and anti-SSB-La occurred only in 2.3% (n = 5) of the patients. These patients failed to fulfill the AECG criteria. Two (2) showed LSGB focus score of ≥ 1 and diagnosis of SS based on the specialist’s opinion; 3 showed both biopsy result and specialist’s opinion negative for SS.

Comparing patients with pSS (AECG) and nonspecific dryness syndrome, the pSS patients were older, presented more severe salivary and lachrymal dysfunction, more frequency of ANA antibodies, anti-SSA-Ro, and anti-SSB-La; and had more systemic manifestations. Fibromyalgia was the most prevalent comorbidity among patients with pSS. Of the 98 patients with fibromyalgia and who were referred because of sicca syndrome, 36.7% (n = 36) displayed LSGB compatible with SS (focus score of ≥1) (Table 2).

**Table 2:** **Clinical characteristics of patients with primary Sjögren’s syndrome and nonspecific dryness syndrome**

	**Primary Sjögren’s syndrome (AECG) n = 65**	**Nonspecific dryness syndrome n = 132**	*p-value
Age (years)	49.6 ± 12	45.8 ± 12.9	*0.02*
Gender (Women)	65 (98.5%)	123 (94.6%)	0.36
Oral symptoms	60 (90.9%)	95 (85.6%)	0.42
Ocular symptoms	61 (92.4%)	90 (81.1%)	0.07
Salivary flow (<0.1 ml/min)	53 (80.3%)	51 (46.4%)	*0.00*
Schirmer (≤5 mm)	42 (70.0%)	50 (56.2%)	0.13
Focal Sialoadenitis (focus score ≥ 1)	59 (89.4%)	7 (5.4%)	*0.00*
Anti-SSA and/or anti-SSB	21 (32.3%)	6 (6.2%)	*0.00*
Rheumatoid Factor (látex)	14 (25.9%)	22 (21.0%)	0.62
ANA	39 (66.1%)	44 (40.0%)	*0.00*
Fibromyalgia	41 (63%)	57 (43.6%)	*0.01*
Hypertension	26 (39.4%)	38 (29.2%)	0.20
Dyslipidemia	10 (15.2%)	12 (9.2%)	0.31
Diabetes Mellitus	4 (6.1%)	13 (10.0%)	0.52
Depression	8 (12.1%)	12 (9.2%)	0.70
Hyperthyroidism, Graves Disease	8 (12.1%)	9 (6.9%)	0.34
Osteoporosis/Osteopenia	8 (12.1%)	9 (6.9%)	0.34
Osteoarthritis	20 (30.3%)	28 (21.5%)	0.24
Neoplasia	5 (7.6%)	2 (1.5%)	0.08

*Chi-square Test.

## Discussion

This study was about the relevance of LSGB for diagnosis of pSS in real life, analyzing a cohort of patients referred for biopsy. Only a few published studies have evaluated the diagnostic usefulness of MSGB in pSS [8]. Most studies were on retrospective analysis, and evaluation of biopsy accuracy was not the main objective. Our study compared LSGB results with a re-evaluation conducted by 2 specialists; high sensibility, specificity, and accuracy were found. The only study with comparable methodology also found high sensibility (85.7%), specificity (89.7%), PPV (85.7%) and NPV (89.7%) [9]. In others studies, sensibility and specificity of LSGB ranged from 63.5% to 93.7% and from 61.2% to 100%, respectively [8]. It seems that specificity and PPV are high, and sensitivity is variable depending on the profile of the studied patients.

The diagnosis of pSS is not easy, and it is guided by a combination of clinical manifestations, glandular dysfunction, laboratory exams, and MSGB. At present, MSGB has a major role being included in the AECG 2002 [6] and ACR 2012 criteria [8]. It is also important for the prognosis of SS by giving information about subsets of patients [21]. However, it is an invasive method, and when to indicate in the clinical practice remains to be the difficult decision to make. There is no algorithm or any recommendation about that. Ultrasound is being evaluated to be included in a set of criteria for diagnosis [22-24]. In spite of being a promising tool for diagnosis and prognosis, and having sensibility comparable to scintigraphy and sialography, it is probable that the US could not substitute biopsy for all patients [16,22-24].

We have analyzed the reasons of indicating and the profile of suspected patients referred for MSGB. Dryness occurred in SS in 75.7% to 96.7% [25-27], and it was the most common reason for indicating biopsy in our cohort. However, almost half of the patients had extra-glandular symptoms. In our study there were some patients that had systemic symptoms only. In spite of not being so common, SS should be considered without dryness symptoms, in special in patients with peripheral neuropathy, parotiditis, arthritis, hematological manifestations, or both positive RF and ANA. Another study reported the same extra-glandular manifestation in patients referred for biopsy [17].

It appears that glandular dysfunction measured by USF and ST was a good screening for the diagnosis. Most patients with glandular dysfunction and positive serology (positive anti-SSA-Ro and/or anti-SSB-La) had positive biopsy (≥1 focus score), and those with normal glandular function and negative serology had negative biopsy. LSGB was necessary to define diagnosis in those patients with glandular dysfunction and negative serology. Based on our results, patients with glandular dysfunction and incomplete AECG criteria should be submitted to LSGB, as it is the only way to discriminate patients with or without SS. Consensus recommendation to standardize an investigation algorithm might contribute in increasing the number of diagnosed cases.

In our cohort referred for LSGB, we found 38.9% of SS (30.1% pSS and 8.8% sSS). The frequency of SS in suspected cases using the AECG criteria varied from 32.8% to 79.2% [7,9,28]. Unfortunately, we could not perform a comparison to ACR 2012 criteria because data were collected between 2008 and 2011. Also, we have used just Schirmer’s test in screening dry eyes because it is simple and it can be performed by a rheumatologist. In our clinic, patients are referred to an ophthalmologist only when SS diagnosis is confirmed or when there is a suspected case not fulfilling the criteria. Not including Schirmer’s test in criteria setting is a disadvantage because it is very easy, cheap, and also has sensibility, specificity, and accuracy comparable to other methods (29).

The frequencies of ANA and RF found in our study were 20% and 66.1%, respectively. These rates were similar to results found in previous studies [14,27-30]. The presence of FAN and RF was included in the diagnosis criteria suggested by Fox et al., in 1986 [30], made part of the preliminary criteria proposed by Vitali et al., in 1993 [31], and was inserted again in the criteria proposed by ACR 2012. In our study, concomitant presence of ANA ≥ 1/320 and positive RF in patients without anti-SSA-Ro and anti-SSB-La antibodies occurred in only 2.3% of the patients.

Comparing patients with dryness with or without SS in our study, we noticed a high prevalence of FM in both pSS (63%) and non-SS (43.8%) groups. Moreover, 36.7% of patients with fibromyalgia referred for LSGB because dryness symptoms displayed LSGB with focus score of ≥ 1 and they met the AECG criteria for SS. These data suggest that fibromyalgia is a trick, as differentiating it from diffuse muscle skeletal pain and fatigue of SS is not possible, thus underestimating FM diagnosis in SS. On the other hand, dryness and fatigue are common symptoms in FM patients. Our data suggest that SS should be investigated in patients with FM and dryness.

We did not have any data about complications in our patients. This information was not collected from all patients. Some complications like pain, bruising, bleeding, and wound infection have been reported, but they are rare. The main LSGB complication is lip numbness, but permanent neurological complication brought about by the linear incision technique occurs only in 1.4% of the patients [32]. From our perspective and not based on data, it seems to be a safe technique when performed by experienced professionals. In our hospital, rheumatologists do biopsy and discuss weekly the results with pathologists. Good connections between rheumatologists, pathologists, and professionals doing biopsy, can minimize the risks and optimize the quality and size of the material.

This study was a retrospective study that could result in having bias. For example, in the routine, patients usually are evaluated according to the classification criteria 2002. Also, a specialist’s opinion was considered the gold-standard and biopsy results were considered come up with a final diagnosis. Future prospective studies comparing with ultrasound will be useful.

## Conclusions

Labial salivary gland biopsy has high sensibility, specificity, and positive and negative predictive values. In the clinical practice, it is useful especially for those patients with glandular dysfunctions and negative antibodies.
